# Identifying Risk Groups in 401,846 Osteoarthritis Patients Undergoing Total Hip Arthroplasty: A Machine Learning Clustering Analysis

**DOI:** 10.3390/jpm16060280

**Published:** 2026-05-24

**Authors:** Alishah Ahmadi, Anthony J. Kaywood, Areeb Ansari, Alejandra Chavarria, Oserekpamen Favour Omobhude, Adam Kiss, Mateusz Faltyn, Jason S. Hoellwarth

**Affiliations:** 1 School of Medicine, New York Medical College, Valhalla, NY 10595, USA; akaywood@student.nymc.edu (A.J.K.); achavarr@student.nymc.edu (A.C.); oomobhud@student.nymc.edu (O.F.O.); akiss@student.nymc.edu (A.K.); 2 Department of Biology, St. John’s University, Queens, New York, NY 11439, USA; areeb.ansari793@gmail.com; 3 Research & Development, Trilemma Capital, Vancouver, BC V6Z 2X7, Canada; matt@trilemmacapital.com; 4 Department of Limb Lengthening and Complex Reconstruction, Hospital for Special Surgery, New York, NY 10021, USA

**Keywords:** total hip arthroplasty, osteoarthritis, comorbidities, national inpatient sample, perioperative outcomes

## Abstract

**Background/Objective**: Osteoarthritis (OA) is the most common indication for total hip arthroplasty (THA), yet postoperative utilization and discharge outcomes vary substantially due to heterogeneous comorbidity burdens. This study applied unsupervised machine learning clustering to identify distinct comorbidity profiles among OA patients undergoing THA and to evaluate their association with non-routine discharge (NRD) and length of stay (LOS). **Methods**: The 2015–2021 National Inpatient Sample was queried using ICD-10 CM/PCS codes to identify patients with OA undergoing THA. Forty-nine comorbidities, complications, and in-hospital clinical covariates were incorporated into a k-modes clustering analysis. The Davies–Bouldin and Calinski–Harabasz indices were used to determine the optimal number of clusters. Multivariable logistic regression assessed adjusted odds of NRD across clusters, and Kruskal–Wallis H testing evaluated differences in LOS. **Results**: A total of 401,846 patients were included, and five distinct clusters were identified, ranging from 777 to 331,755 patients. Clusters with higher prevalence of renal dysfunction, cardiovascular disease, anemia, and heart failure demonstrated significantly increased risk of NRD (adjusted odds ratios up to 3.01, *p* < 0.001) and prolonged hospitalization, with median LOS up to 4 days. Lower-risk clusters exhibited shorter hospitalizations with median LOS of 2 days and higher rates of routine discharge. Kruskal–Wallis testing confirmed significant LOS differences across all clusters (*p* < 0.001). **Conclusions**: Machine learning clustering of OA patients undergoing THA identified clinically distinct subgroups with graded differences in postoperative hospital utilization. Patients with greater comorbidity burden experienced disproportionately higher risk of NRD and prolonged LOS. This data-driven framework highlights heterogeneity within the OA population and may inform future strategies for perioperative risk stratification and resource planning.

## 1. Introduction

Osteoarthritis (OA) is a progressive degenerative joint disease characterized by the breakdown of joint tissues over time, leading to chronic pain, stiffness, and reduced mobility [1,2]. OA affects over 32 million people in the United States [2,3] and is most commonly seen in adults over 50 and post-menopausal women, though younger individuals may also develop OA as a result of previous joint injuries, abnormal joint structure, or genetic cartilage defects [4]. Treatment options range from conservative therapies including exercise, corticosteroid injections, and supplements, to surgical intervention for more advanced disease [5,6,7]. Total hip arthroplasty (THA) provides reliable pain relief, restoration of joint function, and improved quality of life for patients with advanced degenerative hip OA [8]. Additionally, outpatient care plays a crucial role in managing OA; one Swedish study demonstrated that individuals with OA had 20 more hospitalizations per 10,000 person-years for ambulatory care-sensitive conditions compared with those without OA [9].

The rise of artificial intelligence, particularly machine learning (ML), has transformed clinical data analysis across multiple disciplines. ML algorithms process high-dimensional patient data to support diagnosis, risk prediction, and treatment planning [10]. In orthopedics, ML has shown strong accuracy in predicting outcomes such as length of stay, discharge disposition, and procedural cost, underscoring its value in perioperative optimization [11]. ML techniques fall into two broad categories: supervised learning, which includes classification and regression, and unsupervised learning, which includes clustering and dimensionality reduction [10,12]. While supervised learning is frequently used for diagnostic or prognostic tasks, unsupervised learning is increasingly employed to identify disease phenotypes and uncover patterns not easily detected through traditional statistical approaches [13]. In OA research, techniques such as UMAP combined with unsupervised clustering have identified clinically distinct phenotypes with differing progression patterns, demonstrating the value of ML in understanding OA heterogeneity [14].

Despite these advances, relatively little is known about how chronic health conditions cluster among patients with OA—particularly those undergoing THA. OA is commonly associated with conditions such as hypertension and depression, but the broader comorbidity patterns in this population remain poorly defined, limiting the precision of preoperative risk assessment [15]. Moreover, prior work applying clustering methods to arthroplasty populations has highlighted the importance of identifying latent comorbidity structures that influence postoperative outcomes [16]. Clustering approaches may therefore provide a more comprehensive understanding of patient risk profiles by uncovering naturally occurring subgroups based on comorbidities, demographics, and other health characteristics that traditional regression methods often fail to capture.

This study applies unsupervised machine learning clustering techniques to a large national cohort of OA patients undergoing THA from the National Inpatient Sample (NIS). The goal is to identify comorbidity-based clusters within this population and determine their associations with postoperative outcomes, including non-routine discharge (NRD) and length of stay (LOS). Prior research has shown that individuals with multiple chronic medical conditions are more likely to experience postoperative complications and prolonged recovery following THA [17]. As interest grows in precision-based perioperative planning, clustering offers a data-driven framework for stratifying risk and improving preoperative optimization. By uncovering clinically meaningful comorbidity patterns among OA patients undergoing THA, this study aims to support more personalized surgical planning and enhance the overall quality of perioperative care.

## 2. Materials and Methods

### 2.1. Data Source

This analysis was conducted utilizing data sourced from the 2015 (Q4)–2021 National Inpatient Sample (NIS) Database which was sponsored by the Agency for Healthcare Research and Quality (AHRQ) and developed for the Healthcare Cost and Utilization Project (HCUP). The NIS is the largest publicly available all-payer database on inpatient stays in the United States; its design approximates a 20% stratified sample of all admissions from long-term acute care hospitals and community hospitals.

### 2.2. Study Design and Population

This investigation was conducted as a retrospective study incorporating clustering analysis to evaluate patients with osteoarthritis undergoing total hip arthroplasty (THA). Hospital admissions meeting study criteria were extracted using both diagnostic and procedural coding from the International Classification of Diseases, Tenth Revision, Clinical Modification and Procedure Coding System (ICD-10-CM and ICD-10-PCS). These codes were used to systematically identify individuals with osteoarthritis, as well as those who underwent THA during the same hospitalization. Hospitalizations including at least one of the procedure codes [‘0SR9019’, ‘0SR901A’, ‘0SR901Z’, ‘0SR9029’, ‘0SR902A’, ‘0SR902Z’, ‘0SR9039’, ‘0SR903A’, ‘0SR903Z’, ‘0SR9049’, ‘0SR904A’, ‘0SR904Z’, ‘0SR9069’, ‘0SR906A’, ‘0SR906Z’, ‘0SR907Z’, ‘0SR90EZ’, ‘0SR90J9’, ‘0SR90JA’, ‘0SR90JZ’, ‘0SR90KZ’, ‘0SRB019’, ‘0SRB01A’, ‘0SRB01Z’, ‘0SRB029’, ‘0SRB02A’, ‘0SRB02Z’, ‘0SRB039’, ‘0SRB03A’, ‘0SRB03Z’, ‘0SRB049’, ‘0SRB04A’, ‘0SRB04Z’, ‘0SRB069’, ‘0SRB06A’, ‘0SRB06Z’, ‘0SRB07Z’, ‘0SRB0EZ’, ‘0SRB0J9’, ‘0SRB0JA’, ‘0SRB0JZ’, ‘0SRB0KZ’] between 2015 Q4 and 2021 were included in the study population.

### 2.3. Data Variables and Outcomes

The demographic characteristics evaluated in this study included patient age, sex, and race (categorized as White, Black, Hispanic, Asian or Pacific Islander, Native American, and Other), along with median household income quartile determined by ZIP code and primary expected payer (Medicare, Medicaid, private insurance, self-pay, no charge, and other).

Clustering was performed using a total of 49 variables encompassing clinical comorbidities, in-hospital complications, and additional inpatient covariates. These variables were derived from diagnosis codes, procedure codes, and the Elixhauser Comorbidity indicators (CMR_xxx). The specific coding definitions used to construct each clustering variable are detailed in Supplementary Table S1, and the complete set of variables is as follows:

Acute Ischemic Stroke (AIS), Acute Respiratory Distress Syndrome (ARDS), Acute Kidney Failure, Alcohol Abuse, Anemia, Anxiety, Arrhythmia, Aspiration Pneumonitis, Autoimmune Disease, COPD, Cancer, Chronic Kidney Disease, Chronic Liver Disease, Coagulopathy, Deep Vein Thrombosis (DVT), Dementia, Dependent (Control), Depression, Diabetes, Drug Abuse, Dysphagia, HIV, Heart Failure, Hydrocephalus, Hypercalcemia, Hyperlipidemia, Hypertension, Hypothyroid, Hypocalcemia, Hyperthyroid, IBD, LowVitD, MI, Mechanical Ventilation, Obesity, Osteoporosis, Prosthetic Joint Infection (PJI), Peripheral Vascular Disease, Surgical Wound Infection, Pulmonary Embolism, Rheumatoid Arthritis, Seizures, Sleep Apnea, Smoking, Tracheostomy, UTI, hemorrhage, pneumonia, sepsis, Wound Dehiscence.

Outcomes analyzed were non-routine discharge (NRD) and length-of-stay (LOS). NRD was defined by values other than 1 in the column indicating patient disposition “DISPUNIFORM”, where a value of 1 indicates a routine discharge. Length-of-stay was a continuous variable extracted from the “LOS” column of the NIS database.

### 2.4. Clustering

To characterize distinct patterns of comorbidities and inpatient covariates within the study population, clustering was conducted using the k-modes unsupervised machine learning algorithm. The k-modes approach was chosen due to its suitability for categorical data and its computational efficiency when applied to large administrative datasets such as the NIS.

**Figure 1:**
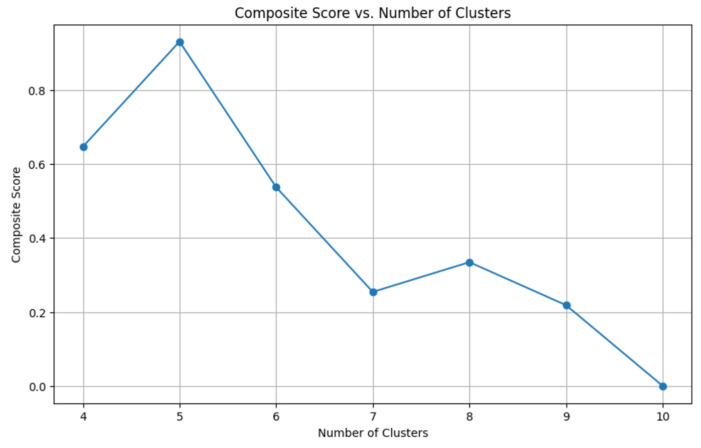
Composite DBI-CHI Scoring for Determination of Optimal Number of Clusters.

The optimal cluster solution was identified as five clusters based on a combined assessment of the Davies–Bouldin Index (DBI) and Calinski–Harabasz Index (CHI). Specifically, an inverted and normalized DBI score was integrated with a normalized CHI score to generate a composite metric. As shown in Figure 1, composite DBI–CHI scores were plotted across cluster solutions ranging from 4 to 10. The resulting curve demonstrates a clear peak at five clusters, supporting this configuration as the most appropriate according to internal validity measures. The composite score rises from 0.65 at four clusters to a maximum of 0.93 at five clusters, followed by a marked decline thereafter.

### 2.5. Statistical Analysis

Descriptive analyses were conducted to summarize demographic characteristics for the overall cohort as well as within each identified cluster, allowing for assessment of distributional trends. Cluster composition was further visualized using a heatmap illustrating the mean prevalence of each clustering variable across clusters. Demographic features for each cluster were also tabulated to facilitate comparison of patterns between groups. When appropriate, pairwise comparisons of comorbidities and inpatient covariates across clusters were performed using Chi-square and Fisher’s exact tests to evaluate statistically significant differences in prevalence.

To examine differences in non-routine discharge (NRD), multivariable logistic regression models were constructed to estimate adjusted odds ratios with corresponding 95% confidence intervals (95% CI) for each cluster relative to Cluster 1. Covariates included in the adjustment were age, sex, race, income quartile, and primary payer type. Unadjusted regression results were additionally reported for comparison.

**Figure 2:**
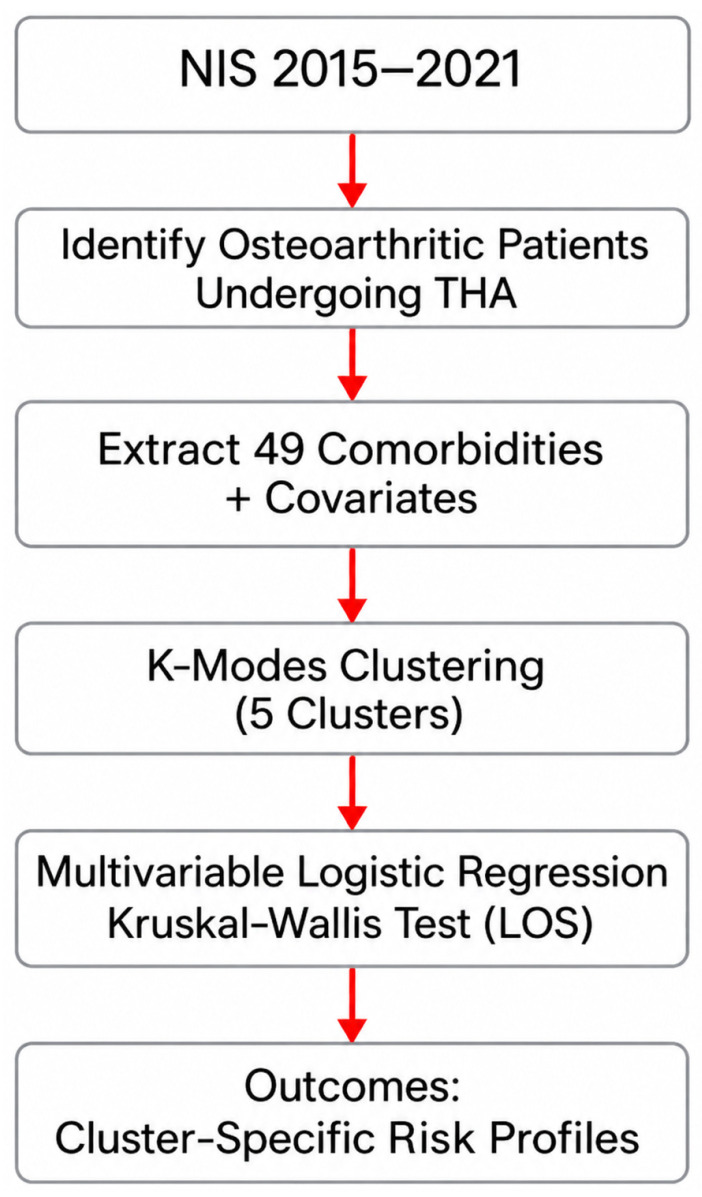
Methodological pipeline illustrating data extraction, clustering, and outcome analysis.

Given the non-parametric nature of length of stay, differences in median values across clusters were assessed using the Kruskal–Wallis H test, as shown in Figure 2. Subsequent post hoc pairwise comparisons were conducted with Bonferroni correction to identify specific inter-cluster differences. The distribution of length of stay was visualized using box-and-whisker plots, violin plots, and density plots. Additional descriptive measures, including the median, first quartile (Q1), third quartile (Q3), and interquartile range, were calculated to further characterize variability across clusters. All statistical analyses were performed in Python (version 3.12.7) utilizing the pandas, matplotlib, k-modes, pyarrow, seaborn, and scikit-learn libraries.

## 3. Results

A total of 401,846 patients with osteoarthritis undergoing total hip arthroplasty (THA) were included in the study cohort. The mean age was 66.10 years with a standard deviation of 10.96 years. Regarding sex distribution, 56.14% of patients identified as female (*n* = 225,595), while 43.86% were male (*n* = 176,251).

As shown in Table 1, patients were distributed across income quartiles as follows: 19.85% (*n* = 79,762) in the first quartile (lowest income), 24.98% (*n* = 100,366) in the second quartile, 26.94% (*n* = 108,265) in the third quartile, and 28.23% (*n* = 113,453) in the fourth quartile (highest income). Medicare was the most common primary payer (55.72%, *n* = 223,911), followed by private insurance (36.15%, *n* = 145,276) and Medicaid (4.91%, *n* = 19,726). Smaller proportions of patients were categorized as self-pay (0.68%, *n* = 2719), no charge (0.06%, *n* = 226), or other payer types (2.49%, *n* = 9988).

By race, the majority of patients were White (85.63%, *n* = 344,116), followed by Black (7.86%, *n* = 31,585) and Hispanic (3.60%, *n* = 14,452) individuals. Asian or Pacific Islander patients accounted for 0.90% of the cohort (*n* = 3631), while Native American patients represented 0.30% (*n* = 1189). An additional 1.71% (*n* = 6873) were categorized as other races.

### 3.1. Clustering Comorbidities

**Figure 3:**
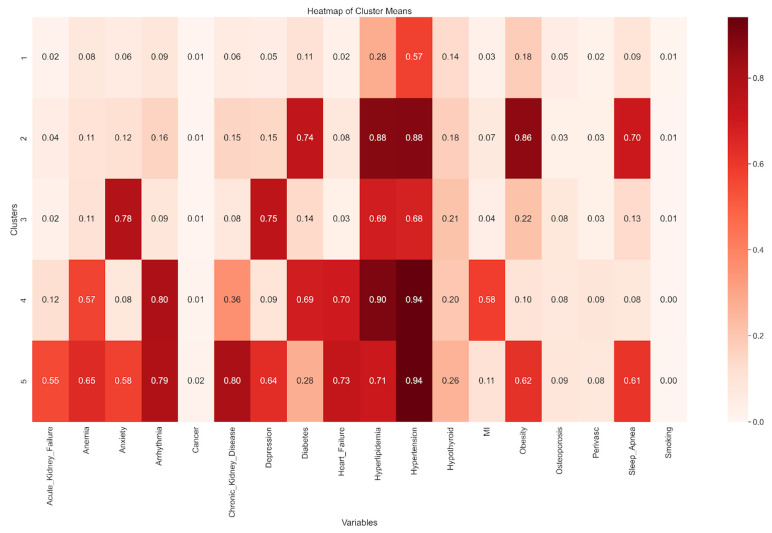
Heatmap indicating mean prevalence of each comorbidity/covariate within clusters 1–5; clusters are numbered by increasing rates of non-routine discharge (NRD). MI = myocardial infarction.

By selection criteria, all patients had osteoarthritis. Across the five clusters generated, each group demonstrated distinct comorbidity patterns that correspond to varying levels of medical complexity. As illustrated in Figure 3, Cluster 1 was characterized by generally low comorbidity burdens, with uniformly low prevalence rates across nearly all measured conditions, identifying it as the healthiest baseline group. Cluster 2 had the highest levels of diabetes, obesity, and sleep apnea. Cluster 3 demonstrated the highest prevalence of anxiety and depression within the cohort. Cluster 4 exhibited a substantially more complex clinical profile, with the highest rates of arrhythmia, hyperlipidemia, and myocardial infarction (MI), as well as notable elevations in hypertension and chronic kidney disease. Finally, Cluster 5 represented the most medically vulnerable group, displaying the highest prevalence of acute kidney failure, chronic kidney disease, anemia, and heart failure. This pattern suggests significant multi-organ dysfunction and advanced cardiometabolic disease, consistent with this cluster’s markedly worse postoperative outcomes, including longer length of stay and increased likelihood of non-routine discharge.

### 3.2. Non-Routine Discharge

The primary aim of this study was to use ML to identify clinically distinct subgroups of osteoarthritic patients undergoing THA based on comorbidity and clinical profiles, and to examine differences in adverse event risk across these groups. As shown in Table 2, multivariable logistic regression examined the association between cluster membership and the odds of NRD, using Cluster 1 (*n* = 331,755) as the reference group. Cluster 2 (*n* = 25,944) demonstrated a modest elevation in unadjusted NRD risk (OR: 1.18, 95% CI: 1.15–1.21; *p* < 0.001), which remained significant after adjustment (aOR: 1.21, 95% CI: 1.18–1.24; *p* < 0.001). Cluster 3 (*n* = 41,110) similarly showed higher crude odds of NRD (OR: 1.25, 95% CI: 1.23–1.28; *p* < 0.001), with the association persisting in the adjusted model (aOR: 1.22, 95% CI: 1.19–1.25; *p* < 0.001). In contrast, Cluster 4 (*n* = 2260) exhibited a markedly increased risk, with both unadjusted (OR: 2.49, 95% CI: 2.26–2.76; *p* < 0.001) and adjusted estimates (aOR: 2.00, 95% CI: 1.81–2.22; *p* < 0.001) indicating substantially higher odds of NRD. Notably, Cluster 5 (*n* = 777) demonstrated the greatest risk elevation among all groups, with an unadjusted OR of 3.56 (95% CI: 2.94–4.31; *p* < 0.001) and an adjusted OR of 3.01 (95% CI: 2.48–3.65; *p* < 0.001), reflecting more than a threefold increase in NRD risk relative to the reference cluster. These findings indicate a clear gradient of risk across cluster groups, with Clusters 4 and 5 representing subpopulations with disproportionately elevated likelihood of non-routine discharge, independent of age, sex, race, income quartile, and payer type.

### 3.3. Length of Stay

**Figure 4:**
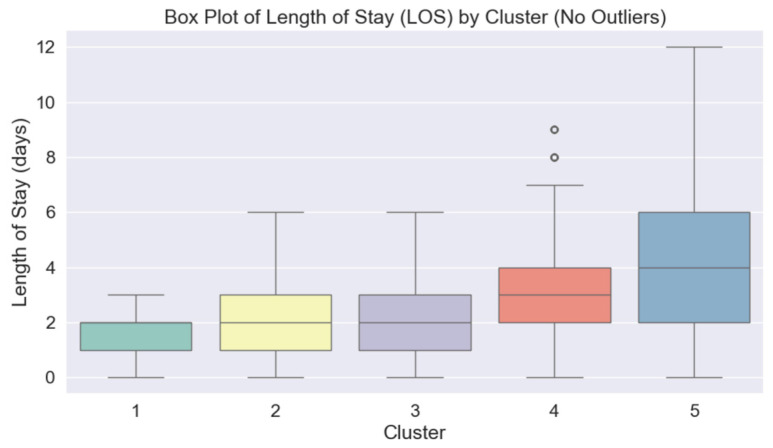
Box-plot comparing the distribution of length of stay (LOS) across all five cluster groups.

Another outcome evaluated in this study was postoperative length of stay (LOS) across the five cluster groups. As shown in Figure 4 and detailed in Table 3, Clusters 1, 2, and 3 each demonstrated a median LOS of 2 days, with narrow interquartile ranges (IQR = 1, 2, and 2, respectively), indicating relatively short and consistent hospitalization durations within these groups. Cluster 4 showed a modest increase, with a median LOS of 3 days (IQR = 3), reflecting greater variability and a slightly prolonged recovery period. In contrast, Cluster 5 exhibited the longest hospitalization course, with a median LOS of 4 days and the widest interquartile range (IQR = 4), suggesting substantially greater heterogeneity and overall longer postoperative recovery among patients in this subgroup.

## 4. Discussion

In this study, we applied an unsupervised machine learning approach to characterize heterogeneity among 401,846 osteoarthritis (OA) patients undergoing total hip arthroplasty (THA) within the National Inpatient Sample. Using the k-modes clustering algorithm, patients were grouped according to shared comorbidity patterns across 49 clinical variables, resulting in five distinct phenotypic clusters with markedly different postoperative utilization profiles. A clear gradient in outcomes emerged across clusters, closely aligning with increasing medical complexity. Patients in Cluster 1, defined mostly by hypertension, demonstrated the shortest median length of stay (LOS) and the lowest likelihood of non-routine discharge (NRD), whereas Cluster 5, defined by their high prevalence of acute kidney failure, chronic kidney disease, heart failure, anemia, and arrhythmia, exhibited the longest median LOS and more than a three-fold increase in NRD compared to the reference group. Importantly, LOS and discharge disposition are best interpreted as indicators of hospital resource utilization and care complexity rather than direct measures of surgical morbidity or recovery quality, as supported by prior arthroplasty outcomes literature [18,19,20].

Examination of cluster-level comorbidity profiles provides important clinical context for these findings. Cluster 5 represented the most medically vulnerable subgroup, characterized by high prevalence of acute kidney failure, chronic kidney disease, heart failure, anemia, and arrhythmia, and demonstrated the greatest postoperative resource demand [21,22]. Cluster 4 similarly exhibited substantial medical complexity, with elevated rates of cardiovascular and renal disease and a two-fold increase in adjusted NRD risk. In contrast, Clusters 2 and 3 demonstrated intermediate utilization patterns, with modest but statistically significant increases in NRD risk and LOS relative to Cluster 1. Notably, mental health emerged as a clinically relevant dimension of postoperative trajectories, particularly in cluster 3 with the highest prevalence of anxiety and depression. Prospective cohort studies and systematic reviews demonstrate that psychiatric conditions are associated with inferior functional outcomes, reduced activity levels, and increased healthcare utilization following THA [23,24,25,26]. Anxiety and depressive symptoms may influence pain perception, motivation for rehabilitation, and recovery expectations, underscoring the importance of incorporating psychological health into perioperative risk stratification and interpretation of utilization-based outcomes.

These findings align with the existing orthopedic literature demonstrating that multimorbidity—particularly involving cardiovascular, renal, and hematologic disease—is a major driver of prolonged hospitalization and discharge to post-acute care following THA. Large observational and registry-based studies have consistently identified renal disease, heart failure, anemia, diabetes, and advanced age as independent predictors of longer LOS and non-home discharge following elective hip arthroplasty [9,27]. These associations persist despite advances in perioperative optimization pathways, underscoring the dominant influence of baseline patient complexity on postoperative care needs.

Beyond confirming established associations, this study highlights the value of unsupervised clustering for identifying latent patient phenotypes that extend beyond traditional risk stratification. Rather than evaluating comorbidities in isolation, clustering captures the combined and interacting effects of multiple chronic conditions on perioperative outcomes. Systematic reviews and methodological analyses emphasize that machine learning approaches can effectively model complex interactions among patient factors and predict outcomes relevant to recovery, utilization, and quality of life after total joint arthroplasty [28,29,30]. Importantly, unsupervised methods such as clustering offer complementary value by identifying clinically interpretable subgroups rather than generating opaque risk scores, thereby supporting more nuanced perioperative planning and targeted resource allocation [29,30]. This work builds directly on previously published clustering analysis in diabetic patients undergoing THA, which similarly demonstrated graded differences in NRD and LOS across comorbidity-defined phenotypes, supporting the generalizability of unsupervised phenotyping across major THA populations [16]. In addition, identification of lower-risk phenotypes, such as Cluster 1, may have implications for surgical planning, including consideration of bone-preserving implant strategies such as short-stem total hip arthroplasty in appropriately selected patients, which may offer advantages in preserving femoral bone stock and facilitating future revision [31].

The interpretation of NRD and LOS warrants careful emphasis. Although frequently used as outcome metrics, these outcomes primarily reflect care coordination demands, rehabilitation needs, and safe discharge planning rather than surgical success or failure. Medically complex patients may require longer hospitalization or discharge to post-acute facilities despite achieving excellent pain relief and functional improvement after THA, emphasizing the need to contextualize utilization-based outcomes within broader frameworks of patient safety and recovery support [20,32].

Several limitations should be acknowledged. This analysis is observational and does not permit causal inference regarding the relationship between comorbidity patterns and postoperative utilization. The National Inpatient Sample lacks granular clinical detail, including laboratory values, baseline functional status, frailty indices, operative time, implant characteristics, and rehabilitation protocols. Additionally, longitudinal outcomes beyond hospital discharge are not captured. For example, because the National Inpatient Sample is a discharge-level database without unique patient identifiers, patients undergoing staged bilateral total hip arthroplasty during separate admissions may have been counted as independent encounters rather than as a single individual. Comorbidities were identified using administrative coding, introducing potential misclassification bias. Although the NIS provides a large nationally derived inpatient sample, it remains an administrative discharge-level database subject to coding variability, sampling limitations, and limited perioperative clinical granularity. Accordingly, the findings should be interpreted within the context of the HCUP/AHRQ sampling framework and the exploratory nature of the clustering analysis. Finally, although Cluster 5 represented a relatively small subgroup, unsupervised clustering approaches may identify rare but clinically important phenotypes characterized by disproportionately elevated comorbidity burden and healthcare utilization. Nevertheless, the smaller sample size of this cluster relative to lower-risk groups may limit the stability and generalizability of these estimates.

Future research should validate these phenotypic clusters in datasets with greater clinical resolution, including joint replacement registries or linked electronic health record systems that incorporate laboratory values, functional measures, perioperative variables, and post-discharge outcomes. Prospective evaluation is needed to determine whether early identification of high-risk phenotypes can guide targeted preoperative optimization, perioperative monitoring, and discharge planning strategies [33,34,35]. Integrating such strategies with machine learning–based phenotyping may enable more personalized, efficient, and equitable perioperative care pathways for OA patients undergoing THA.

## 5. Conclusions

This study utilized an unsupervised machine learning clustering approach to stratify a nationally representative cohort of patients with osteoarthritis undergoing total hip arthroplasty, identifying five clinically distinct subgroups with heterogeneous comorbidity profiles and differing levels of hospital resource utilization. Patients within the highest-risk clusters—particularly those characterized by renal dysfunction, cardiovascular disease, anemia, and heart failure—demonstrated substantially higher rates of non-routine discharge and prolonged hospitalization, whereas lower-risk clusters exhibited shorter lengths of stay and more favorable discharge patterns. These findings highlight the marked heterogeneity within the osteoarthritis population and demonstrate how unsupervised clustering can reveal latent comorbidity structures associated with perioperative utilization that are not readily apparent through traditional analytic approaches. However, these results should be interpreted cautiously and not applied directly to individual clinical decision-making. Given the reliance on administrative data without granular perioperative variables, functional measures, or longitudinal follow-up, further investigation is necessary to determine whether these clusters correspond to meaningful differences in long-term clinical outcomes. Future studies incorporating prospective validation, laboratory and functional data, and post-discharge outcomes are needed to assess whether machine learning–derived phenotypes can be integrated into perioperative risk stratification and care planning. At present, this work provides a data-driven foundation for future efforts aimed at refining risk assessment and resource allocation among osteoarthritis patients undergoing total hip arthroplasty.

## Figures and Tables

**Table 1:** Demographics of patients undergoing total hip arthroplasty (THA) with osteoarthritis.

Sample Size		Age (Years)		Sex	*n* (%)
Total	401,846	Mean	66.10	Male	176,251 (43.86)
		St. Dev.	10.96	Female	225,595 (56.14)
**Income Quartile**	***n* (%)**	**Payer Type**	***n* (%)**	**Race**	***n* (%)**
1st	79,762 (19.85)	Medicare	223,911 (55.72)	White	344,116 (85.63)
2nd	100,366 (24.98)	Medicaid	19,726 (4.91)	Black	31,585 (7.86)
3rd	108,265 (26.94)	Private insurance	145,276 (36.15)	Hispanic	14,452 (3.6)
4th	113,453 (28.23)	Self-pay	2719 (0.68)	Asian or Pacific Islander	3631 (0.9)
		No charge	226 (0.06)	Native American	1189 (0.3)
		Other	9988 (2.49)	Other	6873 (1.71)

**Table 2:** Multivariable Logistic Regression of Non-Routine Discharge with Cluster 1 as the reference group. Age, sex, race, income quartile and payer type are controlled for in the aORs.

Cluster #	Cluster Size	Odds Ratio (95% CI)	*p*-Value	Adjusted Odds Ratio (95% CI)	*p*-Value
1	331,755	ref	ref	ref	ref
2	25,944	1.18 (1.15–1.21)	<0.001	1.21 (1.18–1.24)	<0.001
3	41,110	1.25 (1.23–1.28)	<0.001	1.22 (1.19–1.25)	<0.001
4	2260	2.49 (2.26–2.76)	<0.001	2.00 (1.81–2.22)	<0.001
5	777	3.56 (2.94–4.31)	<0.001	3.01 (2.48–3.65)	<0.001

**Table 3:** Median number of days, first quartile, third quartile, and interquartile ranges for each cluster group.

Cluster	Median Days Spent Hospitalized Post-Surgery	Q1	Q3	IQR
1	2	1	2	1
2	2	1	3	2
3	2	1	3	2
4	3	2	5	3
5	4	2	6	4

## Data Availability

The original contributions presented in this study are included in the article/Supplementary Material. Further inquiries can be directed to the corresponding author.

## Supplementary Materials

The following supporting information can be downloaded at https://www.mdpi.com/article/10.3390/jpm16060280/s1, Figure S1. Heatmap indicating mean prevalence of all comorbidity/covariate within clusters 1–6; Clusters are numbered by increasing rates of non-routine discharge, Table S1. Inclusion criteria and all variables along with ICD-10 codes analyzed for each cluster group.

## References

[1] Kolasinski S.L., Neogi T., Hochberg M.C., Oatis C., Guyatt G., Block J., Callahan L., Copenhaver C., Dodge C., Felson D. (2020). 2019 American College of Rheumatology/Arthritis Foundation Guideline for the Management of Osteoarthritis of the Hand, Hip, and Knee. Arthritis Rheumatol..

[2] GBD 2021 Osteoarthritis Collaborators (2023). Global, regional, and national burden of osteoarthritis, 1990–2020 and projections to 2050: A systematic analysis for the Global Burden of Disease Study 2021. Lancet Rheumatol..

[3] Katz J.N., Arant K.R., Loeser R.F. (2021). Diagnosis and Treatment of Hip and Knee Osteoarthritis: A Review. JAMA.

[4] Sen R., Hurley J.A. (2025). Osteoarthritis. StatPearls [Internet].

[5] Sinusas K. (2012). Osteoarthritis: Diagnosis and treatment. Am. Fam. Physician.

[6] Burns R.B., Skorupa T., Abdeen A., Kanjee Z. (2025). What Would You Recommend for This Patient Interested in a Total Knee Joint Arthroplasty? Grand Rounds Discussion From Beth Israel Deaconess Medical Center. Ann. Intern. Med..

[7] Hannon C.P., Goodman S.M., Austin M.S., Yates A., Guyatt G., Aggarwal V.K., Baker J.F., Bass P., Bekele D.I., Dass D. (2023). 2023 American College of Rheumatology and American Association of Hip and Knee Surgeons clinical practice guideline for the optimal timing of elective hip or knee arthroplasty for patients with symptomatic moderate-to-severe osteoarthritis or advanced symptomatic osteonecrosis with secondary arthritis for whom nonoperative therapy is ineffective. Arthritis Care Res..

[8] Varacallo M.A., Luo T.D., Johanson N.A. (2025). Total Hip Arthroplasty Techniques. Updated 4 August 2023. StatPearls [Internet].

[9] Kiadaliri A., Englund M. (2021). Osteoarthritis and risk of hospitalization for ambulatory care-sensitive conditions: A general population-based cohort study. Rheumatology.

[10] Ngiam K.Y., Khor I.W. (2019). Big data and machine learning algorithms for health-care delivery. Lancet Oncol..

[11] Lalehzarian S.P., Gowd A.K., Liu J.N. (2021). Machine learning in orthopaedic surgery. World J. Orthop..

[12] Ono S., Goto T. (2022). Introduction to supervised machine learning in clinical epidemiology. Ann. Clin. Epidemiol..

[13] Esteva A., Robicquet A., Ramsundar B., Kuleshov V., DePristo M., Chou K., Cui C., Corrado G., Thrun S., Dean J. (2019). A guide to deep learning in healthcare. Nat. Med..

[14] Huang Z., Guo W., Martin J. (2022). Clustering and dimensional reduction for visualizing knee osteoarthritis phenotypes: Data from the OAI. Osteoarthr. Cartil..

[15] Swain S., Coupland C., Strauss V., Mallen C., Kuo C.F., Sarmanova A., Bierma-Zeinstra S.M.A., Englund M., Prieto-Alhambra D., Doherty M. (2022). Clustering of comorbidities and associated outcomes in people with osteoarthritis—A UK Clinical Practice Research Datalink study. Osteoarthr. Cartil..

[16] Ahmadi A., Kaywood A.J., Chavarria A., Omobhude O.F., Kiss A., Faltyn M., Hoellwarth J.S. (2025). Identifying Risk Groups in 73,000 Patients with Diabetes Receiving Total Hip Replacement: A Machine Learning Clustering Analysis. J. Pers. Med..

[17] Belmont PJJr Goodman G.P., Waterman B.R., Bader J.O., Schoenfeld A.J. (2014). Thirty-day postoperative complications and mortality following total knee arthroplasty: Incidence and risk factors among a national sample of 15,321 patients. J. Bone Jt. Surg. Am..

[18] Sibia U.S., MacDonald J.H., King P.J. (2016). Predictors of hospital length of stay in an enhanced recovery after surgery program for primary total hip arthroplasty. J. Arthroplast..

[19] Flanigan T.L., Kiskaddon E.M., Rogozinski J.A., Thomas M.D., Froehle A.W., Krishnamurthy A.B. (2021). Predictive factors of extended length of hospital stay following total joint arthroplasty in a Veterans Affairs hospital population. J. Arthroplast..

[20] Viswanathan V.K., Subramanian S., Jones H., Mounasamy V., Sambandam S. (2024). Patient disposition after discharge following primary total hip arthroplasty: Home versus skilled nursing facility. Arch. Orthop. Trauma Surg..

[21] Mannion A.F., Nauer S., Arsoy D., Impellizzeri F.M., Leunig M. (2020). The association between comorbidity and the risks and early benefits of total hip arthroplasty for hip osteoarthritis. J. Arthroplast..

[22] Podmore B., Hutchings A., Skinner J.A., MacGregor A.J., van der Meulen J. (2021). Impact of comorbidities on the safety and effectiveness of hip and knee arthroplasty surgery. Bone Jt. J..

[23] Aalders M.B., Ligthart M.J., Temmerman O.P., Benner J.L., van der List J.P., Kerkhoffs G.M., Keijser L.C. (2025). Anxiety and depression symptoms are associated with inferior outcomes in patients undergoing total hip arthroplasty. J. Arthroplast..

[24] Hecht C.J., Burkhart R.J., Karimi A.H., Acuña A.J., Kamath A.F. (2023). Association between psychiatric illness and total joint arthroplasty outcomes. Clin. Orthop. Relat. Res..

[25] Reine S., Xi Y., Archer H., Chhabra A., Huo M., Wells J. (2023). Effects of depression, anxiety, and pain catastrophizing on total hip arthroplasty patient activity level. J. Arthroplast..

[26] Duivenvoorden T., Vissers M., Verhaar J., Busschbach J., Gosens T., Bloem R., Bierma-Zeinstra S., Reijman M. (2013). Anxiety and depressive symptoms before and after total hip and knee arthroplasty. Osteoarthr. Cartil..

[27] Li G., Yu F., Liu S., Weng J., Qi T., Qin H., Chen Y., Wang F., Xiong A., Wang D. (2023). Patient characteristics and procedural variables are associated with length of stay and hospital cost among unilateral primary total hip arthroplasty patients: A single-center retrospective cohort study. BMC Musculoskelet. Disord..

[28] Karlin E.A., Lin C.C., Meftah M., Slover J.D., Schwarzkopf R. (2023). The impact of machine learning on total joint arthroplasty patient outcomes: A systematic review. J. Arthroplast..

[29] Polce E.M., Kunze K.N., Dooley M.S., Piuzzi N.S., Boettner F., Sculco P.K. (2022). Efficacy and applications of artificial intelligence and machine learning analyses in total joint arthroplasty. J. Bone Jt. Surg. Am..

[30] Hunter J., Soleymani F., Viktor H., Michalowski W., Poitras S., Beaulé P.E. (2024). Using unsupervised machine learning to predict quality of life after total knee arthroplasty. J. Arthroplast..

[31] Melišík M., Hrubina M., Heřt J., Cibula Z., Čabala J., Nečas L. (2021). Strednedobé výsledky ultra-krátkeho anatomického drieku Proxima: Analýza 130 prípadov [Mid-Term Results of Proxima Ultra-Short Anatomical Stem: Analysis of 130 Cases]. Acta Chir. Orthop. Traumatol. Cech..

[32] Kim K.Y., Anoushiravani A.A., Chen K.K., Li R., Bosco J.A., Slover J.D., Iorio R. (2019). Perioperative orthopedic surgical home: Optimizing total joint arthroplasty candidates and preventing readmission. J. Arthroplast..

[33] Clarke Z.H., Springer B.D., Sporer S.M., Ast M.P., Chen A.F., Fricka K.B. (2025). Patient preoperative optimization: How to do it and how to be paid for the work. J. Arthroplast..

[34] Johns W.L., Layon D., Golladay G.J., Kates S.L., Scott M., Patel N.K. (2020). Preoperative risk factor screening protocols in total joint arthroplasty. J. Arthroplast..

[35] Forslund J.M., Chan P.H., Prentice H.A., Purdy A.C., Khatod M. (2023). Preoperative patient optimization outcomes from elective total joint arthroplasty. J. Am. Acad. Orthop. Surg..

