# Supplementary material for: Identifying Risk Groups in 401,846 Osteoarthritis Patients Undergoing Total Hip Arthroplasty: A Machine Learning Clustering Analysis
Source: J Pers Med. 2026 May 24;16(6):280. doi: 10.3390/jpm16060280 (PMC13302313; doi:10.3390/jpm16060280)
Supplement: Supplementary file 1 [file jpm-16-00280-s001.zip › jpm-4222426-supplementary.pdf]

Article

Identifying Risk Groups in 401,846 Osteoarthritis Patients Undergoing Total Hip Arthroplasty: A Machine Learning Clustering Analysis

Alishah Ahmadi [1,*], Anthony J. Kaywood [1], Areeb Ansari [2], Alejandra Chavarria [1], Oserekpamen Favour Omobhude [1], Adam Kiss [1], Mateusz Faltyn [3] and Jason S. Hoellwarth [4]

Supplementary

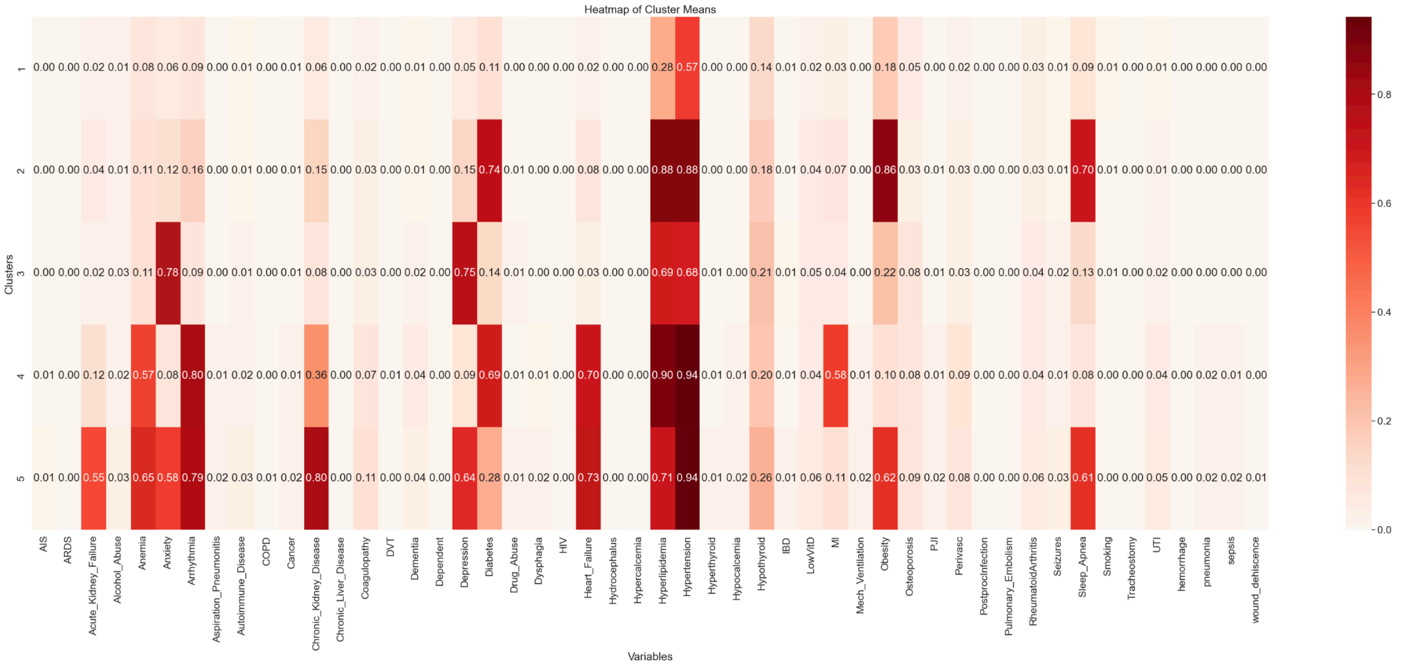

Figure S1. Heatmap indicating mean prevalence of all comorbidity/covariate within clusters 1-6; Clusters are numbered by increasing rates of non-routine discharge.

Table S1. Inclusion criteria and all variables along with ICD-10 codes analyzed for each cluster group.

**Inclusion Criteria**	
	At least one Hospitalization including at least one of the procedure codes ['0SR9019', '0SR901A', '0SR901Z', '0SR9029', '0SR902A', '0SR902Z', '0SR9039', '0SR903A', '0SR903Z', '0SR9049', '0SR904A', '0SR904Z', '0SR9069', '0SR906A', '0SR906Z', '0SR907Z', '0SR90EZ', '0SR90J9', '0SR90JA', '0SR90JZ', '0SR90KZ', '0SRB019', '0SRB01A', '0SRB01Z', '0SRB029', '0SRB02A', '0SRB02Z', '0SRB039', '0SRB03A', '0SRB03Z', '0SRB049', '0SRB04A', '0SRB04Z', '0SRB069', '0SRB06A', '0SRB06Z', '0SRB07Z', '0SRB0EZ', '0SRB0J9', '0SRB0JA', '0SRB0JZ', '0SRB0KZ'] between 2015 Q4 and 2019 were included in the study population.

Clustering Variables	
Alcohol Abuse	CMR_ALCOHOL; F10.1; F101; F10.10; F1010; F10.11; F1011; F10.12; F1012; F10.120; F10120; F10.121; F10121; F10.129; F10129; F10.13; F1013; F10.130; F10130; F10.131; F10131; F10.132; F10132; F10.139; F10139; F10.14; F1014; F10.15; F1015; F10.150; F10150; F10.151; F10151; F10.159; F10159; F10.18; F1018; F10.180; F10180; F10.181; F10181; F10.182; F10182; F10.188; F10188; F10.19; F1019; F10.2; F102; F10.20; F1020; F10.21; F1021; F10.22; F1022; F10.220; F10220; F10.221; F10221; F10.229; F10229; F10.23; F1023; F10.230; F10230; F10.231; F10231; F10.232; F10232; F10.239; F10239; F10.24; F1024; F10.25; F1025; F10.250; F10250; F10.251; F10251; F10.259; F10259; F10.26; F1026; F10.27; F1027; F10.28; F1028; F10.280; F10280; F10.281; F10281; F10.282; F10282; F10.288; F10288; F10.29; F1029; F10.9; F109; F10.90; F1090; F10.91; F1091; F10.92; F1092; F10.920; F10920; F10.921; F10921; F10.929; F10929; F10.93; F1093; F10.930; F10930; F10.931; F10931; F10.932; F10932; F10.939; F10939; F10.94; F1094; F10.95; F1095; F10.950; F10950; F10.951; F10951; F10.959; F10959; F10.96; F1096; F10.97; F1097; F10.98; F1098; F10.980; F10980; F10.981; F10981; F10.982; F10982; F10.988; F10988; F10.99; F1099
Autoimmune Disease	CMR_AUTOIMMUNE
Dementia	CMR_DEMENTIA; F01.5; F015; F01.50; F0150; F01.51; F0151; F01.511; F01511; F01.518; F01518; F01.52; F0152; F01.53; F0153; F01.54; F0154; F01.A; F01A; F01.A0; F01A0; F01.A1; F01A1; F01.A11; F01A11; F01.A18; F01A18; F01.A2; F01A2; F01.A3; F01A3; F01.A4; F01A4; F01.B; F01B; F01.B0; F01B0; F01.B1; F01B1; F01.B11; F01B11; F01.B18; F01B18; F01.B2; F01B2; F01.B3; F01B3; F01.B4; F01B4; F01.C; F01C; F01.C0; F01C0; F01.C1; F01C1; F01.C11; F01C11; F01.C18; F01C18; F01.C2; F01C2; F01.C3; F01C3; F01.C4; F01C4; F02.8; F028; F02.80; F0280; F02.81; F0281; F02.811; F02811; F02.818; F02818; F02.82; F0282; F02.83; F0283; F02.84; F0284; F02.A; F02A; F02.A0; F02A0; F02.A1; F02A1; F02.A11; F02A11; F02.A18; F02A18; F02.A2; F02A2; F02.A3; F02A3; F02.A4; F02A4; F02.B; F02B; F02.B0; F02B0; F02.B1; F02B1; F02.B11; F02B11; F02.B18; F02B18; F02.B2; F02B2; F02.B3; F02B3; F02.B4; F02B4; F02.C; F02C; F02.C0; F02C0; F02.C1; F02C1; F02.C11; F02C11; F02.C18; F02C18; F02.C2; F02C2; F02.C3; F02C3; F02.C4; F02C4; F03.9; F039; F03.90; F0390; F03.91; F0391; F03.911; F03911; F03.918; F03918; F03.92; F0392; F03.93; F0393; F03.94; F0394; F03.A; F03A; F03.A0; F03A0; F03.A1; F03A1; F03.A11; F03A11; F03.A18; F03A18; F03.A2; F03A2; F03.A3; F03A3; F03.A4; F03A4; F03.B; F03B; F03.B0; F03B0; F03.B1; F03B1; F03.B11; F03B11; F03.B18; F03B18; F03.B2; F03B2; F03.B3; F03B3; F03.B4; F03B4; F03.C; F03C; F03.C0; F03C0; F03.C1; F03C1; F03.C11; F03C11; F03.C18; F03C18; F03.C2; F03C2; F03.C3; F03C3; F03.C4; F03C4
Drug Abuse (Opioid, Cannabis, Sedatives, Cocaine, Stimulants, Hallucinogens, Inhalants, Psychoactive Substances, Not including nicotine)	CMR_DRUG_ABUSE; F11.1; F111; F11.10; F1110; F11.11; F1111; F11.12; F1112; F11.120; F11120; F11.121; F11121; F11.122; F11122; F11.129; F11129; F11.13; F1113; F11.14; F1114; F11.15; F1115; F11.150; F11150; F11.151; F11151; F11.159; F11159; F11.18; F1118; F11.181; F11181; F11.182; F11182; F11.188; F11188; F11.19; F1119; F12.1; F121; F12.10; F1210; F12.11; F1211; F12.12; F1212; F12.120; F12120; F12.121; F12121; F12.122; F12122; F12.129; F12129; F12.13; F1213; F12.15; F1215; F12.150; F12150; F12.151; F12151; F12.159; F12159; F12.18; F1218; F12.180; F12180; F12.188; F12188; F12.19; F1219; F13.1; F131; F13.10; F1310; F13.11; F1311; F13.12; F1312; F13.120; F13120; F13.121; F13121; F13.129; F13129; F13.13; F1313; F13.130; F13130; F13.131; F13131; F13.132; F13132; F13.139; F13139; F13.14; F1314; F13.15; F1315; F13.150; F13150; F13.151; F13151; F13.159; F13159; F13.18; F1318; F13.180; F13180; F13.181; F13181; F13.182; F13182; F13.188; F13188; F13.19; F1319; F14.1; F141; F14.10; F1410; F14.11; F1411; F14.12; F1412; F14.120; F14120; F14.121; F14121; F14.122; F14122; F14.129; F14129; F14.13; F1413; F14.14; F1414; F14.15; F1415; F14.150; F14150; F14.151; F14151; F14.159; F14159; F14.18; F1418; F14.180; F14180; F14.181; F14181; F14.182; F14182; F14.188; F14188; F14.19; F1419; F15.1; F151; F15.10; F1510; F15.11; F1511; F15.12; F1512; F15.120; F15120; F15.121; F15121; F15.122; F15122; F15.129; F15129; F15.13; F1513; F15.14; F1514; F15.15; F1515; F15.150; F15150; F15.151; F15151; F15.159; F15159; F15.18; F1518; F15.180; F15180; F15.181; F15181; F15.182; F15182; F15.188; F15188; F15.19; F1519; F16.1; F161; F16.10; F1610; F16.11; F1611; F16.12; F1612; F16.120; F16120; F16.121; F16121; F16.122; F16122; F16.129; F16129; F16.14; F1614; F16.15; F1615; F16.150; F16150; F16.151; F16151; F16.159; F16159; F16.18; F1618; F16.180; F16180; F16.183; F16183; F16.188; F16188; F16.19; F1619; F18.1; F181; F18.10; F1810; F18.11; F1811; F18.12; F1812; F18.120; F18120; F18.121; F18121; F18.129; F18129; F18.14; F1814; F18.15; F1815; F18.150; F18150; F18.151; F18151; F18.159; F18159; F18.17; F1817; F18.18; F1818; F18.180; F18180; F18.188; F18188; F18.19; F1819; F19.1; F191; F19.10; F1910; F19.11; F1911; F19.12; F1912; F19.120; F19120; F19.121; F19121; F19.122; F19122; F19.129; F19129; F19.13; F1913; F19.130; F19130; F19.131; F19131; F19.132; F19132; F19.139; F19139; F19.14; F1914; F19.15; F1915; F19.150; F19150; F19.151; F19151; F19.159; F19159; F19.16; F1916; F19.17; F1917; F19.18; F1918; F19.180; F19180; F19.181; F19181; F19.182; F19182; F19.188; F19188; F19.19; F1919
Obesity	CMR_OBESE; E66.01; E6601; E66.09; E6609; E66.1; E661; E66.2; E662; E66.8; E668; E66.9; E669
Peripheral Vascular Disease	CMR_PERIVASC; I73.0; I730; I73.00; I7300; I73.01; I7301; I73.1; I731; I73.8; I738; I73.81; I7381; I73.89; I7389; I73.9; I739

Diabetes	CMR_DIAB_UNCX; CMR_DIAB_CX; E08.0; E080; E08.00; E0800; E08.01; E0801; E08.1; E081; E08.10; E0810; E08.11; E0811; E08.2; E082; E08.21; E0821; E08.22; E0822; E08.29; E0829; E08.3; E083; E08.31; E0831; E08.311; E08311; E08.319; E08319; E08.32; E0832; E08.321; E08321; E08.3211; E083211; E08.3212; E083212; E08.3213; E083213; E08.3219; E083219; E08.329; E08329; E08.3291; E083291; E08.3292; E083292; E08.3293; E083293; E08.3299; E083299; E08.33; E0833; E08.331; E08331; E08.3311; E083311; E08.3312; E083312; E08.3313; E083313; E08.3319; E083319; E08.339; E08339; E08.3391; E083391; E08.3392; E083392; E08.3393; E083393; E08.3399; E083399; E08.34; E0834; E08.341; E08341; E08.3411; E083411; E08.3412; E083412; E08.3413; E083413; E08.3419; E083419; E08.349; E08349; E08.3491; E083491; E08.3492; E083492; E08.3493; E083493; E08.3499; E083499; E08.35; E0835; E08.351; E08351; E08.3511; E083511; E08.3512; E083512; E08.3513; E083513; E08.3519; E083519; E08.352; E08352; E08.3521; E083521; E08.3522; E083522; E08.3523; E083523; E08.3529; E083529; E08.353; E08353; E08.3531; E083531; E08.3532; E083532; E08.3533; E083533; E08.3539; E083539; E08.354; E08354; E08.3541; E083541; E08.3542; E083542; E08.3543; E083543; E08.3549; E083549; E08.355; E08355; E08.3551; E083551; E08.3552; E083552; E08.3553; E083553; E08.3559; E083559; E08.359; E08359; E08.3591; E083591; E08.3592; E083592; E08.3593; E083593; E08.3599; E083599; E08.36; E0836; E08.37; E0837; E08.37X1; E0837X1; E08.37X2; E0837X2; E08.37X3; E0837X3; E08.37X9; E0837X9; E08.39; E0839; E08.4; E084; E08.40; E0840; E08.41; E0841; E08.42; E0842; E08.43; E0843; E08.44; E0844; E08.49; E0849; E08.5; E085; E08.51; E0851; E08.52; E0852; E08.59; E0859; E08.6; E086; E08.61; E0861; E08.610; E08610; E08.618; E08618; E08.62; E0862; E08.620; E08620; E08.621; E08621; E08.622; E08622; E08.628; E08628; E08.63; E0863; E09.630; E09630; E09.638; E09638; E09.64; E0964; E09.641; E09641; E09.649; E09649; E09.65; E0965; E09.69; E0969; E09.8; E098; E09.9; E099; E10.1; E101; E10.10; E1010; E10.11; E1011; E10.2; E102; E10.21; E1021; E10.22; E1022; E10.29; E1029; E10.3; E103; E10.31; E1031; E10.311; E10311; E10.319; E10319; E10.32; E1032; E10.321; E10321; E10.3211; E103211; E10.3212; E103212; E10.3213; E103213; E10.3219; E103219; E10.329; E10329; E10.3291; E103291; E10.3292; E103292; E10.3293; E103293; E10.3299; E103299; E10.33; E1033; E10.331; E10331; E10.3311; E103311; E10.3312; E103312; E10.3313; E103313; E10.3319; E103319; E10.339; E10339; E10.3391; E103391; E10.3392; E103392; E10.3393; E103393; E10.3399; E103399; E10.34; E1034; E10.341; E10341; E10.3411; E103411; E10.3412; E103412; E10.3413; E103413; E10.3419; E103419; E10.349; E10349; E10.3491; E103491; E10.3492; E103492; E10.3493; E103493; E10.3499; E103499; E10.35; E1035; E10.351; E10351; E10.3511; E103511; E10.3512; E103512; E10.3513; E103513; E10.3519; E103519; E10.352; E10352; E10.3521; E103521; E10.3522; E103522; E10.3523; E103523; E10.3529; E103529; E10.353; E10353; E10.3531; E103531; E10.3532; E103532; E10.3533; E103533; E10.3539; E103539; E10.354; E10354; E10.3541; E103541; E10.3542; E103542; E10.3543; E103543; E10.3549; E103549; E10.355; E10355; E10.3551; E103551; E10.3552; E103552; E10.3553; E103553; E10.3559; E103559; E10.359; E10359; E10.3591; E103591; E10.3592; E103592; E10.3593; E103593; E10.3599; E103599; E10.36; E1036; E10.37; E1037; E10.37X1; E1037X1; E10.37X2; E1037X2; E10.37X3; E1037X3; E10.37X9; E1037X9; E10.39; E1039; E10.4; E104; E10.40; E1040; E10.41; E1041; E10.42; E1042; E10.43; E1043; E10.44; E1044; E10.49; E1049; E10.5; E105; E10.51; E1051; E10.52; E1052; E10.59; E1059; E10.6; E106; E10.61; E1061; E10.610; E10610; E10.618; E10618; E10.62; E1062; E10.620; E10620; E10.621; E10621; E10.622; E10622; E10.628; E10628; E10.63; E1063; E10.630; E10630; E10.638; E10638; E10.64; E1064; E10.641; E10641; E10.649; E10649; E10.65; E1065; E10.69; E1069; E10.8; E108; E10.9; E109; E11.0; E110; E11.00; E1100; E11.01; E1101; E11.1; E111; E11.10; E1110; E11.11; E1111; E11.2; E112; E11.21; E1121; E11.22; E1122; E11.29; E1129; E11.3; E113; E11.31; E1131; E11.311; E11311; E11.319; E11319; E11.32; E1132; E11.321; E11321; E11.3211; E113211; E11.3212; E113212; E11.3213; E113213; E11.3219; E113219; E11.329; E11329; E11.3291; E113291; E11.3292; E113292; E11.3293; E113293; E11.3299; E113299; E11.33; E1133; E11.331; E11331; E11.3311; E113311; E11.3312; E113312; E11.3313; E113313; E11.3319; E113319; E11.339; E11339; E11.3391; E113391; E11.3392; E113392; E11.3393; E113393; E11.3399; E113399; E11.34; E1134; E11.341; E11341; E11.3411; E113411; E11.3412; E113412; E11.3413; E113413; E11.3419; E113419; E11.349; E11349; E11.3491; E113491; E11.3492; E113492; E11.3493; E113493; E11.3499; E113499; E11.35; E1135; E11.351; E11351; E11.3511; E113511; E11.3512; E113512; E11.3513; E113513; E11.3519; E113519; E11.352; E11352; E11.3521; E113521; E11.3522; E113522; E11.3523; E113523; E11.3529; E113529; E11.353; E11353; E11.3531; E113531; E11.3532; E113532; E11.3533; E113533; E11.3539; E113539; E11.354; E11354; E11.3541; E113541; E11.3542; E113542; E11.3543; E113543; E11.3549; E113549; E11.355; E11355; E11.3551; E113551; E11.3552; E113552; E11.3553; E113553; E11.3559; E113559; E11.359; E11359; E11.3591; E113591; E11.3592; E113592; E11.3593; E113593; E11.3599; E113599; E11.36; E1136; E11.37; E1137; E11.37X1; E1137X1; E11.37X2; E1137X2; E11.37X3; E1137X3; E11.37X9; E1137X9; E11.39; E1139; E11.4; E114; E11.40; E1140; E11.41; E1141; E11.42; E1142; E11.43; E1143; E11.44; E1144; E11.49; E1149; E11.5; E115; E11.51; E1151; E11.52; E1152; E11.59; E1159; E11.6; E116; E11.61; E1161; E11.610; E11610; E11.618; E11618; E11.62; E1162; E11.620; E11620; E11.621; E11621; E11.622; E11622; E11.628; E11628; E11.63; E1163; E11.630; E11630; E11.638; E11638; E11.64; E1164; E11.641; E11641; E11.649; E11649; E11.65; E1165; E11.69; E1169; E11.8; E118; E11.9; E119; E13.0; E130; E13.00; E1300; E13.01; E1301; E13.1; E131; E13.10; E1310; E13.11; E1311; E13.2; E132; E13.21; E1321; E13.22; E1322; E13.29; E1329; E13.3; E133; E13.31; E1331; E13.311; E13311; E13.319; E13319; E13.32; E1332; E13.321; E13321; E13.3211; E133211; E13.3212; E133212; E13.3213; E133213; E13.3219; E133219; E13.329; E13329; E13.3291; E133291; E13.3292; E133292; E13.3293; E133293; E13.3299; E133299; E13.33; E1333; E13.331; E13331; E13.3311; E133311; E13.3312; E133312; E13.3313; E133313; E13.3319; E133319; E13.339; E13339; E13.3391; E133391; E13.3392; E133392; E13.3393; E133393; E13.3399; E133399; E13.34; E1334; E13.341; E13341; E13.3411; E133411; E13.3412; E133412; E13.3413; E133413; E13.3419; E133419; E13.349; E13349; E13.3491; E133491; E13.3492; E133492; E13.3493; E133493; E13.3499; E133499; E13.35; E1335; E13.351; E13351; E13.3511; E133511; E13.3512; E133512; E13.3513; E133513; E13.3519; E133519; E13.352; E13352; E13.3521; E133521; E13.3522; E133522; E13.3523; E133523; E13.3529; E133529; E13.353; E13353; E13.3531; E133531; E13.3532; E133532; E13.3533; E133533; E13.3539; E133539; E13.354; E13354; E13.3541; E133541; E13.3542; E133542; E13.3543; E133543; E13.3549; E133549; E13.355; E13355; E13.3551; E133551; E13.3552; E133552; E13.3553; E133553; E13.3559; E133559; E13.359; E13359; E13.3591; E133591; E13.3592; E133592; E13.3593; E133593; E13.3599; E133599; E13.36; E1336; E13.37; E1337; E13.37X1; E1337X1; E13.37X2; E1337X2; E13.37X3; E1337X3; E13.37X9; E1337X9; E13.39; E1339; E13.4; E134; E13.40; E1340; E13.41; E1341; E13.42; E1342; E13.43; E1343; E13.44; E1344; E13.49; E1349; E13.5; E135; E13.51; E1351; E13.52; E1352; E13.59; E1359; E13.6; E136; E13.61; E1361; E13.610; E13610; E13.618; E13618; E13.62; E1362; E13.620; E13620; E13.621; E13621; E13.622; E13622; E13.628; E13628; E13.63; E1363; E13.630; E13630; E13.638; E13638; E13.64; E1364; E13.641; E13641; E13.649; E13649; E13.65; E1365; E13.69; E1369; E13.8; E138; E13.9; E139
Hypertension	CMR_HTN_CX; CMR_HTN_UNCX; I10; I11.0; I110; I11.9; I119; I12.0; I120; I12.9; I129; I13.0; I130; I13.1; I131; I13.10; I1310; I13.11; I1311; I13.2; I132; I15.0; I150; I15.1; I151; I15.2; I152; I15.8; I158; I15.9; I159; I16.0; I160; I16.1; I161; I16.9; I169; I1A.0; I1A0
Hyperlipidemia	E78.4; E784; E78.41; E7841; E78.49; E7849; E78.5; E785; E78.2; E782

Cancer	CMR_CANCER_LYMPH; CMR_CANCER_LEUK; CMR_CANCER_METS; CMR_CANCER_NSITU; CMR_CANCER_SOLID
Myocardial Infarction	I25.2; I252; I21.0; I210; I21.01; I2101; I21.02; I2102; I21.09; I2109; I21.1; I211; I21.11; I2111; I21.19; I2119; I21.2; I212; I21.21; I2121; I21.29; I2129; I21.3; I213; I21.4; I214; I21.9; I219; I21.A; I21A; I21.A1; I21A1; I21.A9; I21A9; I21.B; I21B; I22.0; I220; I22.1; I221; I22.2; I222; I22.8; I228; I22.9; I229
Heart Failure	I50.1; I501; I50.2; I502; I50.20; I5020; I50.21; I5021; I50.22; I5022; I50.23; I5023; I50.3; I503; I50.30; I5030; I50.31; I5031; I50.32; I5032; I50.33; I5033; I50.4; I504; I50.40; I5040; I50.41; I5041; I50.42; I5042; I50.43; I5043; I50.8; I508; I50.81; I5081; I50.810; I50810; I50.811; I50811; I50.812; I50812; I50.813; I50813; I50.814; I50814; I50.82; I5082; I50.83; I5083; I50.84; I5084; I50.89; I5089; I50.9; I509
Acute Kidney Failure	N17.0; N170; N17.1; N171; N17.2; N172; N17.8; N178; N17.9; N179; N99.0; N990
Chronic Kidney Disease	N18.1; N181; N18.2; N182; N18.3; N183; N18.30; N1830; N18.31; N1831; N18.32; N1832; N18.4; N184; N18.5; N185; N18.6; N186; N18.9; N189
Pulmonary Embolism	I26.0; I260; I26.01; I2601; I26.02; I2602; I26.09; I2609; I26.9; I269; I26.90; I2690; I26.92; I2692; I26.93; I2693; I26.94; I2694; I26.99; I2699
Chronic Obstructive Pulmonary Disease (COPD)	J41; J42; J43; J44
Smoking (Nicotine Use)	Z72.0; Z720; F17.200; F17200; F17.201; F17201; F17.203; F17203; F17.208; F17208; F17.209; F17209; F17.210; F17210; F17.211; F17211; F17.213; F17213; F17.218; F17218; F17.219; F17219
Deep Vein Thrombosis (DVT)	I82.4; I824; I826; I82.6; I97.89; I9789; T81.72; T8172
Chronic Liver Disease	K76.9; K769
Depression	CMR_DEPRESS; F32.0; F320; F32.1; F321; F32.2; F322; F32.3; F323; F32.4; F324; F32.5; F325; F32.8; F328; F32.81; F3281; F32.89; F3289; F32.9; F329; F32.A; F32A
Anxiety	F41.0; F410; F41.1; F411; F41.2; F412; F41.3; F413; F41.8; F418; F41.9; F419
HIV/AIDS	CMR_AIDS; B20
Dependent Status	Z74.0; Z740; Z74.01; Z7401; Z74.09; Z7409; Z74.1; Z741; Z74.2; Z742; Z74.3; Z743; Z74.8; Z748; Z74.9; Z749
Acute Ischemic Stroke	I63.0; I630; I63.00; I6300; I63.01; I6301; I63.011; I63011; I63.012; I63012; I63.013; I63013; I63.019; I63019; I63.02; I6302; I63.03; I6303; I63.031; I63031; I63.032; I63032; I63.033; I63033; I63.039; I63039; I63.09; I6309; I63.1; I631; I63.10; I6310; I63.11; I6311; I63.111; I63111; I63.112; I63112; I63.113; I63113; I63.119; I63119; I63.12; I6312; I63.13; I6313; I63.131; I63131; I63.132; I63132; I63.133; I63133; I63.139; I63139; I63.19; I6319; I63.2; I632; I63.20; I6320; I63.21; I6321; I63.211; I63211; I63.212; I63212; I63.213; I63213; I63.219; I63219; I63.22; I6322; I63.23; I6323; I63.231; I63231; I63.232; I63232; I63.233; I63233; I63.239; I63239; I63.29; I6329; I63.3; I633; I63.30; I6330; I63.31; I6331; I63.311; I63311; I63.312; I63312; I63.313; I63313; I63.319; I63319; I63.32; I6332; I63.321; I63321; I63.322; I63322; I63.323; I63323; I63.329; I63329; I63.33; I6333; I63.331; I63331; I63.332; I63332; I63.333; I63333; I63.339; I63339; I63.34; I6334; I63.341; I63341; I63.342; I63342; I63.343; I63343; I63.349; I63349; I63.39; I6339; I63.4; I634; I63.40; I6340; I63.41; I6341; I63.411; I63411; I63.412; I63412; I63.413; I63413; I63.419; I63419; I63.42; I6342; I63.421; I63421; I63.422; I63422; I63.423; I63423; I63.429; I63429; I63.43; I6343; I63.431; I63431; I63.432; I63432; I63.433; I63433; I63.439; I63439; I63.44; I6344; I63.441; I63441; I63.442; I63442; I63.443; I63443; I63.449; I63449; I63.49; I6349; I63.5; I635; I63.50; I6350; I63.51; I6351; I63.511; I63511; I63.512; I63512; I63.513; I63513; I63.519; I63519; I63.52; I6352; I63.521; I63521; I63.522; I63522; I63.523; I63523; I63.529; I63529; I63.53; I6353; I63.531; I63531; I63.532; I63532; I63.533; I63533; I63.539; I63539; I63.54; I6354; I63.541; I63541; I63.542; I63542; I63.543; I63543; I63.549; I63549; I63.59; I6359; I63.6; I636; I63.8; I638; I63.81; I6381; I63.89; I6389; I63.9; I639; I67.81; I6781; I67.82; I6782; G45.0; G450; G45.1; G451; G45.2; G452
Anemia	D50; D50.0; D500; D50.1; D501; D50.8; D508; D50.9; D509; D51; D51.0; D510; D51.1; D511; D51.2; D512; D51.3; D513; D51.8; D518; D51.9; D519; D52; D52.0; D520; D52.1; D521; D52.8; D528; D52.9; D529; D53; D53.0; D530; D53.1; D531; D53.2; D532; D53.8; D538; D53.9; D539; D55; D55.0; D550; D55.1; D551; D55.2; D552; D55.21; D5521; D55.29; D5529; D55.3; D553; D55.8; D558; D55.9; D559; D57.0; D570; D57.00; D5700; D57.01; D5701; D57.02; D5702; D57.03; D5703; D57.04; D5704; D57.09; D5709; D58; D58.0; D580; D58.1; D581; D58.2; D582; D58.8; D588; D58.9; D589; D59; D59.0; D590; D59.1; D591; D59.10; D5910; D59.11; D5911; D59.12;

	D5912; D59.13; D5913; D59.19; D5919; D59.2; D592; D59.3; D593; D59.30; D5930; D59.31; D5931; D59.32; D5932; D59.39; D5939; D59.4; D594; D59.5; D595; D59.6; D596; D59.8; D598; D59.9; D599; D60; D60.0; D600; D60.1; D601; D60.8; D608; D60.9; D609; D61; D61.0; D610; D61.01; D6101; D61.02; D6102; D61.09; D6109; D61.1; D611; D61.2; D612; D61.3; D613; D61.8; D618; D61.81; D6181; D61.810; D61810; D61.811; D61811; D61.818; D61818; D61.82; D6182; D61.89; D6189; D61.9; D619; D63; D63.0; D630; D63.1; D631; D63.8; D638; D64; D64.0; D640; D64.1; D641; D64.2; D642; D64.3; D643; D64.4; D644; D64.8; D648; D64.81; D6481; D64.89; D6489; D64.9; D649
Coagulopathy	D65; D66; D67; D68; D68.0; D680; D68.00; D6800; D68.01; D6801; D68.02; D6802; D68.020; D68020; D68.021; D68021; D68.022; D68022; D68.023; D68023; D68.029; D68029; D68.03; D6803; D68.04; D6804; D68.09; D6809; D68.1; D681; D68.2; D682; D68.3; D683; D68.31; D6831; D68.311; D68311; D68.312; D68312; D68.318; D68318; D68.32; D6832; D68.4; D684; D68.5; D685; D68.51; D6851; D68.52; D6852; D68.59; D6859; D68.6; D686; D68.61; D6861; D68.62; D6862; D68.69; D6869; D68.8; D688; D68.9; D689; D69; D69.0; D690; D69.1; D691; D69.2; D692; D69.3; D693; D69.4; D694; D69.41; D6941; D69.42; D6942; D69.49; D6949; D69.5; D695; D69.51; D6951; D69.59; D6959; D69.6; D696; D69.8; D698; D69.9; D699
Hypothyroid	E00.0; E000; E00.1; E001; E00.2; E002; E00.9; E009; E01.0; E010; E01.1; E011; E01.2; E012; E01.8; E018; E02; E03.0; E030; E03.1; E031; E03.2; E032; E03.3; E033; E03.4; E034; E03.5; E035; E03.8; E038; E03.9; E039
Low Vitamin D	'E559'
Non-Life Threatening Arrhythmia	I44; I44.0; I440; I44.1; I441; I44.4; I444; I44.5; I445; I44.7; I447; I45; I45.0; I450; I45.1; I451; I45.10; I4510; I45.19; I4519; I45.2; I452; I45.3; I453; I45.6; I456; I45.8; I458; I45.81; I4581; I45.89; I4589; I47.1; I471; I47.10; I4710; I47.11; I4711; I47.19; I4719; I48.0; I480; I48.1; I481; I48.11; I4811; I48.19; I4819; I48.2; I482; I48.20; I4820; I48.21; I4821; I48.3; I483; I48.4; I484; I48.91; I4891; I49.1; I491; I49.2; I492; I49.3; I493; I49.4; I494; I49.40; I4940; I49.49; I4949; I49.5; I495; I49.8; I498
Sleep Apnea	G47.30; G4730; G47.31; G4731; G47.32; G4732; G47.33; G4733; G47.34; G4734; G47.35; G4735; G47.36; G4736; G47.37; G4737; G47.38; G4738; G47.39; G4739
Inflammatory Bowel Disease (IBD)	K50; K50.0; K500; K50.00; K5000; K50.01; K5001; K50.011; K50011; K50.012; K50012; K50.013; K50013; K50.014; K50014; K50.018; K50018; K50.019; K50019; K50.1; K501; K50.10; K5010; K50.11; K5011; K50.111; K50111; K50.112; K50112; K50.113; K50113; K50.114; K50114; K50.118; K50118; K50.119; K50119; K50.8; K508; K50.80; K5080; K50.81; K5081; K50.811; K50811; K50.812; K50812; K50.813; K50813; K50.814; K50814; K50.818; K50818; K50.819; K50819; K50.9; K509; K50.90; K5090; K50.91; K5091; K50.911; K50911; K50.912; K50912; K50.913; K50913; K50.914; K50914; K50.918; K50918; K50.919; K50919; K51; K51.0; K510; K51.00; K5100; K51.01; K5101; K51.011; K51011; K51.012; K51012; K51.013; K51013; K51.014; K51014; K51.018; K51018; K51.019; K51019; K51.2; K512; K51.20; K5120; K51.21; K5121; K51.211; K51211; K51.212; K51212; K51.213; K51213; K51.214; K51214; K51.218; K51218; K51.219; K51219; K51.3; K513; K51.30; K5130; K51.31; K5131; K51.311; K51311; K51.312; K51312; K51.313; K51313; K51.314; K51314; K51.318; K51318; K51.319; K51319; K51.4; K514; K51.40; K5140; K51.41; K5141; K51.411; K51411; K51.412; K51412; K51.413; K51413; K51.414; K51414; K51.418; K51418; K51.419; K51419; K51.5; K515; K51.50; K5150; K51.51; K5151; K51.511; K51511; K51.512; K51512; K51.513; K51513; K51.514; K51514; K51.518; K51518; K51.519; K51519; K51.8; K518; K51.80; K5180; K51.81; K5181; K51.811; K51811; K51.812; K51812; K51.813; K51813; K51.814; K51814; K51.818; K51818; K51.819; K51819; K51.9; K519; K51.90; K5190; K51.91; K5191; K51.911; K51911; K51.912; K51912; K51.913; K51913; K51.914; K51914; K51.918; K51918; K51.919; K51919

Osteoporosis	M80; M80.0; M800; M80.00; M8000; M80.00XA; M8000XA; M80.00XD; M8000XD; M80.00XG; M8000XG; M80.00XK; M8000XK; M80.00XP; M8000XP; M80.00XS; M8000XS; M80.01; M8001; M80.011; M80011; M80.011A; M80011A; M80.011D; M80011D; M80.011G; M80011G; M80.011K; M80011K; M80.011P; M80011P; M80.011S; M80011S; M80.012; M80012; M80.012A; M80012A; M80.012D; M80012D; M80.012G; M80012G; M80.012K; M80012K; M80.012P; M80012P; M80.012S; M80012S; M80.019; M80019; M80.019A; M80019A; M80.019D; M80019D; M80.019G; M80019G; M80.019K; M80019K; M80.019P; M80019P; M80.019S; M80019S; M80.02; M8002; M80.021; M80021; M80.021A; M80021A; M80.021D; M80021D; M80.021G; M80021G; M80.021K; M80021K; M80.021P; M80021P; M80.021S; M80021S; M80.022; M80022; M80.022A; M80022A; M80.022D; M80022D; M80.022G; M80022G; M80.022K; M80022K; M80.022P; M80022P; M80.022S; M80022S; M80.029; M80029; M80.029A; M80029A; M80.029D; M80029D; M80.029G; M80029G; M80.029K; M80029K; M80.029P; M80029P; M80.029S; M80029S; M80.03; M8003; M80.031; M80031; M80.031A; M80031A; M80.031D; M80031D; M80.031G; M80031G; M80.031K; M80031K; M80.031P; M80031P; M80.031S; M80031S; M80.032; M80032; M80.032A; M80032A; M80.032D; M80032D; M80.032G; M80032G; M80.032K; M80032K; M80.032P; M80032P; M80.032S; M80032S; M80.039; M80039; M80.039A; M80039A; M80.039D; M80039D; M80.039G; M80039G; M80.039K; M80039K; M80.039P; M80039P; M80.039S; M80039S; M80.04; M8004; M80.041; M80041; M80.041A; M80041A; M80.041D; M80041D; M80.041G; M80041G; M80.041K; M80041K; M80.041P; M80041P; M80.041S; M80041S; M80.042; M80042; M80.042A; M80042A; M80.042D; M80042D; M80.042G; M80042G; M80.042K; M80042K; M80.042P; M80042P; M80.042S; M80042S; M80.049; M80049; M80.049A; M80049A; M80.049D; M80049D; M80.049G; M80049G; M80.049K; M80049K; M80.049P; M80049P; M80.049S; M80049S; M80.05; M8005; M80.051; M80051; M80.051A; M80051A; M80.051D; M80051D; M80.051G; M80051G; M80.051K; M80051K; M80.051P; M80051P; M80.051S; M80051S; M80.052; M80052; M80.052A; M80052A; M80.052D; M80052D; M80.052G; M80052G; M80.052K; M80052K; M80.052P; M80052P; M80.052S; M80052S; M80.059; M80059; M80.059A; M80059A; M80.059D; M80059D; M80.059G; M80059G; M80.059K; M80059K; M80.059P; M80059P; M80.059S; M80059S; M80.06; M8006; M80.061; M80061; M80.061A; M80061A; M80.061D; M80061D; M80.061G; M80061G; M80.061K; M80061K; M80.061P; M80061P; M80.061S; M80061S; M80.062; M80062; M80.062A; M80062A; M80.062D; M80062D; M80.062G; M80062G; M80.062K; M80062K; M80.062P; M80062P; M80.062S; M80062S; M80.069; M80069; M80.069A; M80069A; M80.069D; M80069D; M80.069G; M80069G; M80.069K; M80069K; M80.069P; M80069P; M80.069S; M80069S; M80.07; M8007; M80.071; M80071; M80.071A; M80071A; M80.071D; M80071D; M80.071G; M80071G; M80.071K; M80071K; M80.071P; M80071P; M80.071S; M80071S; M80.072; M80072; M80.072A; M80072A; M80.072D; M80072D; M80.072G; M80072G; M80.072K; M80072K; M80.072P; M80072P; M80.072S; M80072S; M80.079; M80079; M80.079A; M80079A; M80.079D; M80079D; M80.079G; M80079G; M80.079K; M80079K; M80.079P; M80079P; M80.079S; M80079S; M80.08; M8008; M80.08XA; M8008XA; M80.08XD; M8008XD; M80.08XG; M8008XG; M80.08XK; M8008XK; M80.08XP; M8008XP; M80.08XS; M8008XS; M80.0A; M800A; M80.0AXA; M800AXA; M80.0AXD; M800AXD; M80.0AXG; M800AXG; M80.0AXK; M800AXK; M80.0AXP; M800AXP; M80.0AXS; M800AXS; M80.0B; M800B; M80.0B1; M800B1; M80.0B1A; M800B1A; M80.0B1D; M800B1D; M80.0B1G; M800B1G; M80.0B1K; M800B1K; M80.0B1P; M800B1P; M80.0B1S; M800B1S; M80.0B2; M800B2; M80.0B2A; M800B2A; M80.0B2D; M800B2D; M80.0B2G; M800B2G; M80.0B2K; M800B2K; M80.0B2P; M800B2P; M80.0B2S; M800B2S; M80.0B9; M800B9; M80.0B9A; M800B9A; M80.0B9D; M800B9D; M80.0B9G; M800B9G; M80.0B9K; M800B9K; M80.0B9P; M800B9P; M80.0B9S; M800B9S; M80.8; M808; M80.80; M8080; M80.80XA; M8080XA; M80.80XD; M8080XD; M80.80XG; M8080XG; M80.80XK; M8080XK; M80.80XP; M8080XP; M80.80XS; M8080XS; M80.81; M8081; M80.811; M80811; M80.811A; M80811A; M80.811D; M80811D; M80.811G; M80811G; M80.811K; M80811K; M80.811P; M80811P; M80.811S; M80811S; M80.812; M80812; M80.812A; M80812A; M80.812D; M80812D; M80.812G; M80812G; M80.812K; M80812K; M80.812P; M80812P; M80.812S; M80812S; M80.819; M80819; M80.819A; M80819A; M80.819D; M80819D; M80.819G; M80819G; M80.819K; M80819K; M80.819P; M80819P; M80.819S; M80819S; M80.82; M8082; M80.821; M80821; M80.821A; M80821A; M80.821D; M80821D; M80.821G; M80821G; M80.821K; M80821K; M80.821P; M80821P; M80.821S; M80821S; M80.822; M80822; M80.822A; M80822A; M80.822D; M80822D; M80.822G; M80822G; M80.822K; M80822K; M80.822P; M80822P; M80.822S; M80822S; M80.829; M80829; M80.829A; M80829A; M80.829D; M80829D; M80.829G; M80829G; M80.829K; M80829K; M80.829P; M80829P; M80.829S; M80829S; M80.83; M8083; M80.831; M80831; M80.831A; M80831A; M80.831D; M80831D; M80.831G; M80831G; M80.831K; M80831K; M80.831P; M80831P; M80.831S; M80831S; M80.832; M80832; M80.832A; M80832A; M80.832D; M80832D; M80.832G; M80832G; M80.832K; M80832K; M80.832P; M80832P; M80.832S; M80832S; M80.839; M80839; M80.839A; M80839A; M80.839D; M80839D; M80.839G; M80839G; M80.839K; M80839K; M80.839P; M80839P; M80.839S; M80839S; M80.84; M8084; M80.841; M80841; M80.841A; M80841A; M80.841D; M80841D; M80.841G; M80841G; M80.841K; M80841K; M80.841P; M80841P; M80.841S; M80841S; M80.842; M80842; M80.842A; M80842A; M80.842D; M80842D; M80.842G; M80842G; M80.842K; M80842K; M80.842P; M80842P; M80.842S; M80842S; M80.849; M80849; M80.849A; M80849A; M80.849D; M80849D; M80.849G; M80849G; M80.849K; M80849K; M80.849P; M80849P; M80.849S; M80849S; M80.85; M8085; M80.851; M80851; M80.851A; M80851A; M80.851D; M80851D; M80.851G; M80851G; M80.851K; M80851K; M80.851P; M80851P; M80.851S; M80851S; M80.852; M80852; M80.852A; M80852A; M80.852D; M80852D; M80.852G; M80852G; M80.852K; M80852K; M80.852P; M80852P; M80.852S; M80852S; M80.859; M80859; M80.859A; M80859A; M80.859D; M80859D; M80.859G; M80859G; M80.859K; M80859K; M80.859P; M80859P; M80.859S; M80859S; M80.86; M8086; M80.861; M80861; M80.861A;

	M80861A; M80.861D; M80861D; M80.861G; M80861G; M80.861K; M80861K; M80.861P; M80861P; M80.861S; M80861S; M80.862; M80862; M80.862A; M80862A; M80.862D; M80862D; M80.862G; M80862G; M80.862K; M80862K; M80.862P; M80862P; M80.862S; M80862S; M80.869; M80869; M80.869A; M80869A; M80.869D; M80869D; M80.869G; M80869G; M80.869K; M80869K; M80.869P; M80869P; M80.869S; M80869S; M80.87; M8087; M80.871; M80871; M80.871A; M80871A; M80.871D; M80871D; M80.871G; M80871G; M80.871K; M80871K; M80.871P; M80871P; M80.871S; M80871S; M80.872; M80872; M80.872A; M80872A; M80.872D; M80872D; M80.872G; M80872G; M80.872K; M80872K; M80.872P; M80872P; M80.872S; M80872S; M80.879; M80879; M80.879A; M80879A; M80.879D; M80879D; M80.879G; M80879G; M80.879K; M80879K; M80.879P; M80879P; M80.879S; M80879S; M80.88; M8088; M80.88XA; M8088XA; M80.88XD; M8088XD; M80.88XG; M8088XG; M80.88XK; M8088XK; M80.88XP; M8088XP; M80.88XS; M8088XS; M80.8A; M808A; M80.8AXA; M808AXA; M80.8AXD; M808AXD; M80.8AXG; M808AXG; M80.8AXK; M808AXK; M80.8AXP; M808AXP; M80.8AXS; M808AXS; M80.8B; M808B; M80.8B1; M808B1; M80.8B1A; M808B1A; M80.8B1D; M808B1D; M80.8B1G; M808B1G; M80.8B1K; M808B1K; M80.8B1P; M808B1P; M80.8B1S; M808B1S; M80.8B2; M808B2; M80.8B2A; M808B2A; M80.8B2D; M808B2D; M80.8B2G; M808B2G; M80.8B2K; M808B2K; M80.8B2P; M808B2P; M80.8B2S; M808B2S; M80.8B9; M808B9; M80.8B9A; M808B9A; M80.8B9D; M808B9D; M80.8B9G; M808B9G; M80.8B9K; M808B9K; M80.8B9P; M808B9P; M80.8B9S; M808B9S; M81; M81.0; M810; M81.6; M816; M81.8; M818
Hypercalcemia	['E8352']
Hypocalcemia	['E8351']
Mechanical Ventilation	['Z99.11'], ['5A1945Z', '5A1955Z', '5A1935Z']
Prosthetic Joint Infection	['T8450XA', 'T8450XD', 'T8450XS', 'T8451XA', 'T8451XD', 'T8451XS', 'T8452XA', 'T8452XD', 'T8452X
Wound dehiscence	T81.3; T813; T81.30; T8130; T81.30XA; T8130XA; T81.30XD; T8130XD; T81.30XS; T8130XS; T81.31; T8131; T81.31XA; T8131XA; T81.31XD; T8131XD; T81.31XS; T8131XS; T81.32; T8132; T81.32XA; T8132XA; T81.32XD; T8132XD; T81.32XS; T8132XS; T81.33; T8133; T81.33XA; T8133XA; T81.33XD; T8133XD; T81.33XS; T8133XS; T81.4; T814; T81.40; T8140; T81.40XA; T8140XA; T81.40XD; T8140XD; T81.40XS; T8140XS; T81.41; T8141; T81.41XA; T8141XA; T81.41XD; T8141XD; T81.41XS; T8141XS; T81.42; T8142; T81.42XA; T8142XA; T81.42XD; T8142XD; T81.42XS; T8142XS; T81.43; T8143; T81.43XA; T8143XA; T81.43XD; T8143XD; T81.43XS; T8143XS; T81.44; T8144; T81.44XA; T8144XA; T81.44XD; T8144XD; T81.44XS; T8144XS; T81.49; T8149; T81.49XA; T8149XA; T81.49XD; T8149XD; T81.49XS; T8149XS
Pneumonia	J95.89; J9589; J13; J14; J17; J15.0; J150; J15.1; J151; J15.2; J152; J15.20; J1520; J15.21; J1521; J15.211; J15211; J15.212; J15212; J15.29; J1529; J15.3; J153; J15.4; J154; J15.5; J155; J15.6; J156; J15.61; J1561; J15.69; J1569; J15.7; J157; J15.8; J158; J15.9; J159; J16.0; J160; J16.8; J168; J18.0; J180; J18.1; J181; J18.2; J182; J18.8; J188; J18.9; J189
Urinary Tract Infection Following a Procedure	N39.0; N390; N99.89; N9989; T83.511; T83511
Acute Respiratory Distress Syndrome (ARDS)	J80; R06.03; R0603
Aspiration Pneumonitis	J69.0; J690; J69.1; J691; J69.8; J698
Dysphagia	R13.1; R131; R13.10; R1310; R13.11; R1311; R13.12; R1312; R13.13; R1313; R13.14; R1314; R13.19; R1319

Seizures with and without Status Epilepticus	G40.0; G400; G40.00; G4000; G40.001; G40001; G40.009; G40009; G40.01; G4001; G40.011; G40011; G40.019; G40019; G40.1; G401; G40.10; G4010; G40.101; G40101; G40.109; G40109; G40.11; G4011; G40.111; G40111; G40.119; G40119; G40.2; G402; G40.20; G4020; G40.201; G40201; G40.209; G40209; G40.21; G4021; G40.211; G40211; G40.212; G40212; G40.213; G40213; G40.219; G40219; G40.3; G403; G40.30; G4030; G40.301; G40301; G40.309; G40309; G40.31; G4031; G40.311; G40311; G40.319; G40319; G40.A; G40A; G40.A0; G40A0; G40.A01; G40A01; G40.A09; G40A09; G40.A1; G40A1; G40.A11; G40A11; G40.A19; G40A19; G40.B; G40B; G40.B0; G40B0; G40.B01; G40B01; G40.B09; G40B09; G40.B1; G40B1; G40.B11; G40B11; G40.B19; G40B19; G40.C; G40C; G40.C0; G40C0; G40.C01; G40C01; G40.C09; G40C09; G40.C1; G40C1; G40.C11; G40C11; G40.C19; G40C19; G40.4; G404; G40.40; G4040; G40.401; G40401; G40.409; G40409; G40.41; G4041; G40.411; G40411; G40.419; G40419; G40.42; G4042; G40.5; G405; G40.50; G4050; G40.501; G40501; G40.509; G40509; G40.8; G408; G40.80; G4080; G40.801; G40801; G40.802; G40802; G40.803; G40803; G40.804; G40804; G40.81; G4081; G40.811; G40811; G40.812; G40812; G40.813; G40813; G40.814; G40814; G40.82; G4082; G40.821; G40821; G40.822; G40822; G40.823; G40823; G40.824; G40824; G40.83; G4083; G40.833; G40833; G40.834; G40834; G40.89; G4089; G40.9; G409; G40.90; G4090; G40.901; G40901; G40.909; G40909; G40.91; G4091; G40.911; G40911; G40.919; G40919
Hydrocephalus	G91.0; G910; G91.1; G911; G91.2; G912; G91.3; G913; G91.4; G914; G91.8; G918; G91.9; G919
Surgical Wound Infection	'T8149', 'T8149XA', 'T8149XD', 'T8149XS', 'T8140', 'T8140XA', 'T8140XD', 'T8140XS', 'T8141', 'T8141XA', 'T8141XD', 'T8141XS', 'T8142', 'T8142XA', 'T8142XD', 'T8142XS', 'T8143', 'T8143XA', 'T8143XD', 'T8143XS', 'T8144', 'T8144XA', 'T8144XD', 'T8144XS']
Rheumatoid Arthritis	['M0560', 'M05611', 'M05612', 'M05619', 'M05621', 'M05622', 'M05629', 'M05631', 'M05632', 'M05639', 'M05641', 'M05642', 'M05649', 'M05651', 'M05652', 'M05659', 'M05661', 'M05662', 'M05669', 'M05671', 'M05672', 'M05679', 'M0569', 'M0570', 'M05711', 'M05712', 'M05719', 'M05721', 'M05722', 'M05729', 'M05731', 'M05732', 'M05739', 'M05741', 'M05742', 'M05749', 'M05751', 'M05752', 'M05759', 'M05761', 'M05762', 'M05769', 'M05771', 'M05772', 'M05779', 'M0579', 'M057A', 'M0580', 'M05811', 'M05812', 'M05819', 'M05821', 'M05822', 'M05829', 'M05831', 'M05832', 'M05839', 'M05841', 'M05842', 'M05849', 'M05851', 'M05852', 'M05859', 'M05861', 'M05862', 'M05869', 'M05871', 'M05872', 'M05879', 'M0589', 'M058A', 'M059', 'M0600', 'M06011', 'M06012', 'M06019', 'M06021', 'M06022', 'M06029', 'M06031', 'M06032', 'M06039', 'M06041', 'M06042', 'M06049', 'M06051', 'M06052', 'M06059', 'M06061', 'M06062', 'M06069', 'M06071', 'M06072', 'M06079', 'M0608', 'M0609', 'M060A', 'M061', 'M0620', 'M06211', 'M06212', 'M06219', 'M06221', 'M06222', 'M06229', 'M06231', 'M06232', 'M06239', 'M06241', 'M06242', 'M06249', 'M06251', 'M06252', 'M06259', 'M06261', 'M06262', 'M06269', 'M06271', 'M06272', 'M06279', 'M0628', 'M0629', 'M0630', 'M06311', 'M06312', 'M06319', 'M06321', 'M06322', 'M06329', 'M06331', 'M06332', 'M06339', 'M06341', 'M06342', 'M06349', 'M06351', 'M06352', 'M06359', 'M06361', 'M06362', 'M06369', 'M06371', 'M06372', 'M06379', 'M0638', 'M0639', 'M064', 'M0680', 'M06811', 'M06812', 'M06819', 'M06821', 'M06822', 'M06829', 'M06831', 'M06832', 'M06839', 'M06841', 'M06842', 'M06849', 'M06851', 'M06852', 'M06859', 'M06861', 'M06862', 'M06869', 'M06871', 'M06872', 'M06879', 'M0688', 'M0689', 'M068A', 'M069', 'M0800', 'M08011', 'M08012', 'M08019', 'M08021', 'M08022', 'M08029', 'M08031', 'M08032', 'M08039', 'M08041', 'M08042', 'M08049', 'M08051', 'M08052', 'M08059', 'M08061', 'M08062', 'M08069', 'M08071', 'M08072', 'M08079', 'M0808', 'M0809', 'M080A', 'M0820', 'M08211', 'M08212', 'M08219', 'M08221', 'M08222', 'M08229', 'M08231', 'M08232', 'M08239', 'M08241', 'M08242', 'M08249', 'M08251', 'M08252', 'M08259', 'M08261', 'M08262', 'M08269', 'M08271', 'M08272', 'M08279', 'M0828', 'M0829', 'M082A', 'M083', 'M0840', 'M08411', 'M08412', 'M08419', 'M08421', 'M08422', 'M08429', 'M08431', 'M08432', 'M08439', 'M08441', 'M08442', 'M08449', 'M08451', 'M08452', 'M08459', 'M08461', 'M08462', 'M08469', 'M08471', 'M08472', 'M08479', 'M0848', 'M084A']
Hyperthyroid	E05.0', 'E050', 'E05.00', 'E0500', 'E05.01', 'E0501', 'E05.1', 'E051', 'E05.10', 'E0510', 'E05.11', 'E0511', 'E05.2', 'E052', 'E05.20', 'E0520', 'E05.21', 'E0521', 'E05.3', 'E053', 'E05.30', 'E0530', 'E05.31', 'E0531', 'E05.4', 'E054', 'E05.40', 'E0540', 'E05.41', 'E0541', 'E05.8', 'E058', 'E05.80', 'E0580', 'E05.81', 'E0581', 'E05.9', 'E059', 'E05.90', 'E0590', 'E05.91', 'E0591'
Tracheostomy	['J95.0', 'J95.00', 'J95.01', 'J95.02', 'J95.03', 'J95.04', 'J95.09', 'Z99.11', 'Z93.0']
Hemorrhage/Hematoma	['L76', 'G97.3', 'G973', 'G97.5', 'G975', 'G97.6', 'G976', 'M96.8', 'M968', 'K91.87', 'K9187', 'K91.84', 'K9184', 'K91.6', 'K916', 'J95.83', 'J9583', 'J95.86', 'J9586', 'I97.4', 'I974', 'I97.5', 'I975', 'I97.6', 'I976']

Sepsis	['A41.0', 'A410', 'A41.01', 'A4101', 'A41.02', 'A4102', 'A41.1', 'A411', 'A41.2', 'A412', 'A41.3', 'A413', 'A41.4', 'A414', 'A41.5', 'A415', 'A41.50', 'A4150', 'A41.51', 'A4151', 'A41.52', 'A4152', 'A41.53', 'A4153', 'A41.54', 'A4154', 'A41.59', 'A4159', 'A41.8', 'A418', 'A41.81', 'A4181', 'A41.89', 'A4189', 'A41.9', 'A419']
